# A Neuron, Microglia, and Astrocyte Triple Co-culture Model to Study Alzheimer’s Disease

**DOI:** 10.3389/fnagi.2022.844534

**Published:** 2022-04-14

**Authors:** Celia Luchena, Jone Zuazo-Ibarra, Jorge Valero, Carlos Matute, Elena Alberdi, Estibaliz Capetillo-Zarate

**Affiliations:** ^1^Department of Neuroscience, University of the Basque Country (UPV/EHU), Leioa, Spain; ^2^Achucarro Basque Center for Neuroscience, Leioa, Spain; ^3^CIBERNED, Centro de Investigación Biomédica en Red Enfermedades Neurodegenerativas, Madrid, Spain; ^4^Institute of Neurosciences of Castilla y León, University of Salamanca, Salamanca, Spain; ^5^Institute for Biomedical Research of Salamanca, Salamanca, Spain; ^6^IKERBASQUE, Basque Foundation for Science, Bilbao, Spain

**Keywords:** neuron, astrocyte, microglia, *in vitro*, co-culture, Alzheimer, synapse, inflammation

## Abstract

Glial cells are essential to understand Alzheimer’s disease (AD) progression, given their role in neuroinflammation and neurodegeneration. There is a need for reliable and easy to manipulate models that allow studying the mechanisms behind neuron and glia communication. Currently available models such as co-cultures require complex methodologies and/or might not be affordable for all laboratories. With this in mind, we aimed to establish a straightforward *in vitro* setting with neurons and glial cells to study AD. We generated and optimized a 2D triple co-culture model with murine astrocytes, neurons and microglia, based on sequential seeding of each cell type. Immunofluorescence, western blot and ELISA techniques were used to characterize the effects of oligomeric Aβ (oAβ) in this model. We found that, in the triple co-culture, microglia increased the expression of anti-inflammatory marker Arginase I, and reduced pro-inflammatory iNOS and IL-1β, compared with microglia alone. Astrocytes reduced expression of pro-inflammatory A1 markers AMIGO2 and C3, and displayed a ramified morphology resembling physiological conditions. Anti-inflammatory marker TGF-β1 was also increased in the triple co-culture. Lastly, neurons increased post-synaptic markers, and developed more and longer branches than in individual primary cultures. Addition of oAβ in the triple co-culture reduced synaptic markers and increased CD11b in microglia, which are hallmarks of AD. Consequently, we developed a straightforward and reproducible triple co-cultured model, where cells resemble physiological conditions better than in individual primary cultures: microglia are less inflammatory, astrocytes are less reactive and neurons display a more mature morphology. Moreover, we are able to recapitulate Aβ-induced synaptic loss and CD11b increase. This model emerges as a powerful tool to study neurodegeneration and neuroinflammation in the context of AD and other neurodegenerative diseases.

## Introduction

Alzheimer’s disease (AD) is a neurodegenerative disease characterized, among other hallmarks, by Amyloid-β (Aβ) peptide aggregation and accumulation, synapse loss and inflammation (Castellani et al., 2010). AD is a multifactorial disease, and thus, understanding the relationship between glial cells and neurons is essential to comprehend the highly complex mechanisms triggered by the accumulation of Aβ.

The use of animal models of AD has demonstrated that targeting glial cells can ameliorate the disease progression (Furman et al., 2012; Czirr et al., 2017). While AD animal models allow researchers to study the complexity of neuropathology and inflammation, mechanistic studies together with cell-to-cell communication analyses are limited due to the inherently large number of variables present in animal models. *In vivo* models might also require long time courses and high financial efforts. Moreover, high number of animals might be required. On the other hand, widely used *in vitro* models such as individual primary cultures, immortalized cell lines and, more recently, human-derived cells are more susceptible to manipulation, time scales are shorter, and the amount of animals required is smaller. However, they lack complexity.

Different co-culture models have been developed in recent years to study the interaction between glial cells and neurons. To study cell communication *via* secreted components, conditioned media have been extensively used. For example, Aβ-stimulated conditioned media from microglia was discovered to stimulate neurotoxicity *via* secreted TNFα and NMDA receptor stimulation in mouse primary neurons (Floden et al., 2005). Secreted factors from astrocytes can also affect neuronal function. Christopherson et al. (2005) found that conditioned media from immature astrocytes contains thrombospondins-1 and -2, which promote synaptogenesis in retinal ganglion cells. In another study, conditioned media from LPS-activated microglia containing IL-1α, TNF, and C1q, induced A1 phenotype in astrocytes, which reduced their capacity to promote neuronal survival and synaptogenesis (Liddelow et al., 2017). Other researchers have developed co-culture models where the two cell populations share the medium but are not in contact with each other. For instance, Du et al. (2018) established an Induced Pluripotent Stem Cells (iPSC)-derived neuron-astrocyte co-culture using transwell inserts, and found that astrocytes rescued mitochondrial dysfunction in dopaminergic neurons treated with mitochondrial toxins. Another advantage of co-cultures is that they can mitigate the drastic transcriptomic modifications that cells, especially microglia, undergo due to manipulation and the modification of their environment. Transcriptomic profiling of human and mouse microglia has shown significant downregulation of genes related to immune signaling and brain development, and upregulation of genes related to inflammation and stress responses in *in vitro* conditions, compared to *ex vivo* (Gosselin et al., 2017).

Microglia *in vitro* and in the brain also have different responses to Aβ. Murine primary microglia display rapid and substantial transcriptional changes in response to Aβ, while *in vivo* microglia do not display the same gene expression changes, suggesting that primary microglia poorly recapitulate *in vivo* conditions (McFarland et al., 2021). Nevertheless, studies have shown that culturing microglia together with neurons can mitigate this problem. iPSC-derived microglia co-cultured with neurons express key microglia-specific markers, display dynamic ramifications, and have phagocytic capacity. They also upregulate homeostatic genes and promote a more anti-inflammatory cytokine profile than monocultures (Haenseler et al., 2017).

In recent years, more complex co-cultures have been developed using three cell populations to study intercellular communication. Park et al. (2018) developed a human triculture model using human neurons, astrocytes, and microglia in a 3D microfluidic platform. With this model, they were able to recapitulate AD features such as Aβ aggregation, tau phosphorylation, and microglial recruitment. They were also able to demonstrate neurotoxic effects derived from neuron-glia interactions (Park et al., 2018). In a different study, a tri-culture system containing human (h)PSC-derived neurons, microglia and astrocytes was used to study complement component C3, and found a reciprocal signaling between microglia and astrocytes that resulted in increased C3 in AD conditions (Guttikonda et al., 2021). However, these models might involve complex methodologies and/or might not be so affordable. In this study, we developed and characterized a murine triple co-culture model with neurons, astrocytes and microglia that can be used to study cellular communication in the context of Aβ pathology and neuroinflammation. It is an inexpensive and straightforward model that better resembles physiological conditions compared to individual primary cultures, and it represents a reproducible *in vitro* model for mechanistic studies of neurodegenerative diseases.

## Materials and Methods

### Animals

Sprague-Dawley wild-type rats of both genders were used to obtain cell cultures and organotypic slices. All animal experiments were compliant with the requirements of the Animal Ethics and Welfare Committee of the University of the Basque Country (UPV/EHU), following the Spanish Real Order 53/2013 and the European Communities Council Directive of September 22nd 2010 (2010/63/EU).

### Cortical Neuron Culture Procedures

Neurons were obtained from the cortical lobes of E18/E19 Sprague-Dawley rat embryos as previously described (Alberdi et al., 2018), with modifications. Cortical lobes were digested in 0.2% trypsin and 0.02% deoxyribonuclease I in Hanks’ Balanced Salts, no calcium, no magnesium (HBSS) (Sigma-Aldrich, MO, United States) for 5 min at 37°C. The enzymatic digestion was stopped by adding Neurobasal medium containing 2% B27 (Gibco, MA, United States), 1% Penicillin-Streptomycin (Lonza, Switzerland) and 0.5 mM L-glutamine, plus 10% fetal bovine serum (FBS) (Gibco, MA, United States). After incubating 10 min at 37°C, cells were centrifuged for 5 min at 200 *g*, and the resulting pellet was resuspended in B27 Neurobasal medium plus 10% FBS. Cells were mechanically dissociated by trituration (20 strokes with 21, 23, and 25-gauge needles), filtered through a 41 μm nylon mesh and seeded onto poly-D-lysine-coated (PDL) (Sigma-Aldrich, MO, United States) plates as needed. The medium was replaced by serum-free B27 Neurobasal 24 h later. Cultures were maintained at 37°C and 5% CO_2_ for 8–9 days *in vitro* (DIV) before use.

### Cortical Glial Culture Procedures

Primary cultures of mixed glial cells (source cultures) were prepared from the cortical lobes of P0/P1 Sprague-Dawley rat pups as described elsewhere (Wyssenbach et al., 2016; Zabala et al., 2018). Cortical lobes were incubated with 0.2% trypsin and 0.01% deoxyribonuclease I in HBSS for 15 min at 37°C. The digestion was stopped adding Iscove’s Modified Dubecco’s Medium (IMDM) (Gibco, MA, United States) with 1% Antibiotic-Antimycotic (Gibco, MA, United States), plus 10% HyClone*™* FBS (Cytiva, MA, United States). Cells were centrifuged for 6 min at 250 *g*, and the pellet was resuspended in IMDM plus 10% HyClone*™* FBS. This pellet was mechanically dissociated by trituration (20 strokes with 21 and 23-gauge needles) and recentrifuged. Cells were resuspended in IMDM plus 10% HyClone*™* FBS and plated onto PDL-coated 75 cm^2^ flasks (about four cortices per flask). One day later, the medium was replaced by fresh IMDM plus 10% HyClone*™* FBS for primary astrocytes, or by Dulbecco’s Modified Eagle Medium high glucose, pyruvate (DMEM) (Gibco, MA, United States) with 1% Penicillin-Streptomycin (Lonza, Switzerland) plus 10% FBS for primary microglia. Cultures were maintained at 37°C and 5% CO_2_ for 1 week to collect astrocytes, or for 2 weeks to collect microglia.

Primary astrocytes were obtained from source mixed glial cultures as described previously (Wyssenbach et al., 2016). After 1 week of incubation, medium was removed, and flasks were washed twice with HBSS. Cells were trypsinized by incubating with Trypsin-ethylenediamine tetraacetic acid solution (EDTA) 0.05% (Gibco, MA, United States) for 5 min at 37°C. IMDM plus 10% HyClone*™* FBS was added to stop the enzymatic reaction and cells were centrifuged for 5 min at 300 *g*. The cell pellet was resuspended in IMDM plus 10% HyClone*™* FBS and astrocytes were plated onto PDL-coated plates as needed. The medium was replaced by serum-free B27 Neurobasal 24 h after plating. Cultures were maintained at 37°C and 5% CO_2_.

Primary microglia were also obtained from source mixed glial cultures as previously described (Zabala et al., 2018). After growing for 2 weeks, microglia were harvested by orbital shaking for 1 h at 35 g in DMEM plus 10% FBS. Cells were centrifuged for 6 min at 250 *g* and plated onto PDL-coated plates as needed. The medium was replaced by serum-free B27 Neurobasal 24 h after plating. Cultures were maintained at 37°C and 5% CO_2_.

### Triple Co-culture Procedures

**FIGURE 1:**
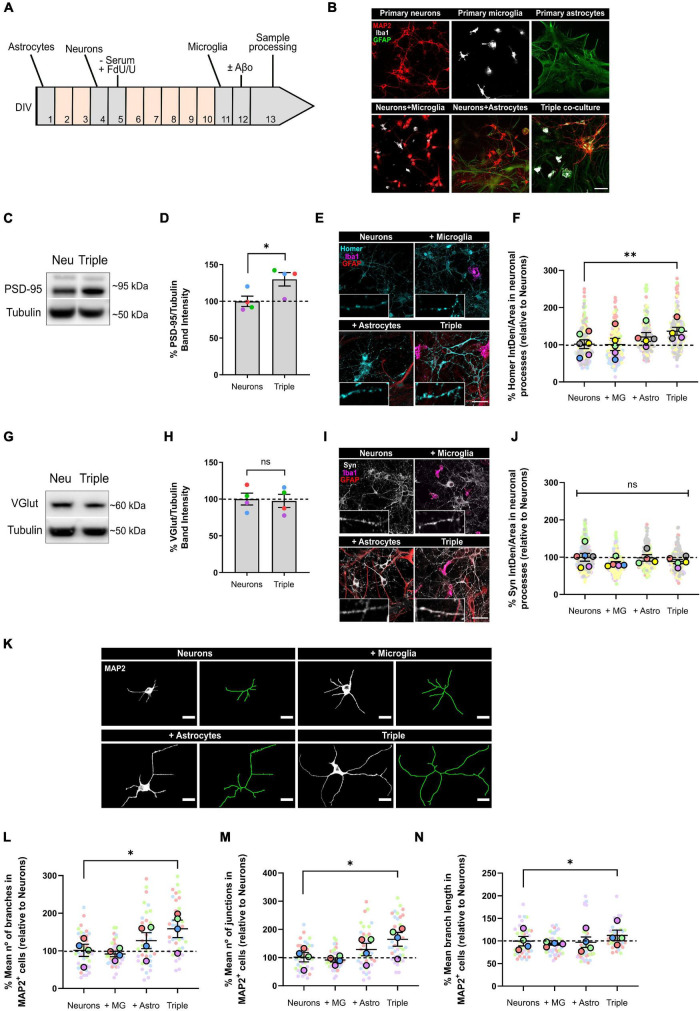
Neurons increased the expression of post-synaptic markers PSD-95 and Homer1, and developed more and longer branches when co-cultured with microglia and astrocytes. **(A)** Protocol diagram for the *in vitro* triple co-culture model and **(B)** representative images. Cultures started with a monolayer of astrocytes. Neurons were plated 3 days later (ratio: five neurons to two astrocytes), and microglia were added 7 days after neurons (ratio: one microglia to five neurons). Cultures were maintained for 13 DIV in total. Scale bar = 50 μm. **(C)** Representative western blot image of post-synaptic marker PSD-95 in total lysates. **(D)** Quantification of PSD-95 in the triple co-culture compared with neurons alone. Raw data was normalized with α-tubulin (*n* = 4). **(E)** Representative images of post-synaptic marker Homer1 immunofluorescence in neuronal processes. Scale bar = 40 μm. **(F)** Quantification of Homer1 in the triple co-culture, compared with neurons alone (*n* = 6). **(G)** Representative western blot image of pre-synaptic marker VGlut1 in total lysates. **(H)** Quantification of VGlu1. Raw data was normalized with α-tubulin (*n* = 4). **(I)** Representative images of pre-synaptic marker Synaptophysin immunofluorescence in neuronal processes. Scale bar = 40 μm. **(J)** Quantification of Synaptophysin (*n* = 6). **(K)** Representative images of the cellular morphology analysis using the neuronal marker MAP2 and the function Skeletonize in Fiji software. Scale bar = 20 μm. **(L)** Quantification of the number of branches per MAP2^+^ cell (*n* = 4). **(M)** Quantification of the number of junctions per MAP2^+^ cell (*n* = 4). **(N)** Mean branch length of MAP2^+^ cells (*n* = 4). Each bar represents the mean ± SEM. *p < 0.05; **p < 0.01; ns, not significant. For quantification, 7–8 random fields per condition were used. DIV = days *in vitro*; Neu, neurons; MG, microglia; Astro, astrocytes; Syn, synaptophysin; IntDen, integrated density.

To obtain all possible combinations of neuron-glia co-cultures (neuron-astrocyte, neuron-microglia and neuron-astrocyte-microglia), primary cortical cell cultures were prepared as stated above. Cells were plated at different ratios as it has been described (Hernangómez et al., 2012; Correa et al., 2013) to find the adequate conditions. The optimal conditions are described in Figure 1A. The co-cultures were started by plating astrocytes onto PDL-coated plates. When a monolayer of astrocytes was obtained 3 days later, neurons were plated in a proportion of five neurons to two astrocytes. After 24 h, the medium was replaced by serum-free B27 Neurobasal, and antimitotic agents 5-Fluoro-2′-deoxyuridine and Uridine (FdU/U) (Sigma-Aldrich, MO, United States) were added at a concentration of 10 μM to control glial proliferation. Cells were incubated at 37°C and 5% CO_2_, and more FdU/U could be added every 2 days for further control of proliferation. Seven days after seeding neurons, microglia was added to the cultures in a proportion of one microglia to five neurons. Cultures were ready to process for protein analysis 2 days later. For experiments with oligomeric Aβ (oAβ), a treatment of 3 μM oAβ for 24 h was used. The co-cultures were kept for a total of 13 DIV at 37°C and 5% CO_2_ in serum-free B27 Neurobasal.

### Conditioned Media Experiments

To obtain conditioned media, primary neurons, astrocytes and microglia were seeded separately at a density of 1 × 10^6^ cells in Petri dishes (60 mm in diameter). One day later, medium was replaced by serum-free B27 Neurobasal, and cells were incubated for 3 days at 37°C and 5% CO_2_. Supernatants were collected, stored at −80°C and filtered with a 22-μm nylon mesh before use.

### Preparation of Soluble Oligomeric Aβ_1–42_

Synthetic Aβ oligomers were prepared as previously reported (Alberdi et al., 2018). Briefly, Aβ_1–42_ (Bachem, Switzerland) was dissolved in hexafluoroisopropanol (HFIP) (Sigma-Aldrich, MO, United States) to an initial concentration of 1 mM. It was distributed into aliquots, and HFIP was removed under vacuum in a speed vac system before the peptide film was stored dried at −80°C. For the aggregation protocol, the peptide was resuspended in anhydrous dimethylsulfoxide (DMSO) (Invitrogen, MA, United States) to a concentration of 5 mM. Hams F-12 pH 7.4 (Biowest, France) was added to reach the final concentration of 100 μM. Oligomers were obtained after incubating at 4°C for 24 h.

### Organotypic Hippocampal Slice Cultures

Hippocampal slice cultures were obtained from P5/P6 Sprague-Dawley rat pups and prepared as previously described (Alberdi et al., 2018; Ortiz-Sanz et al., 2020) with modifications. Brains were extracted in ice cold HBSS, and chopped into 400 μm thick slices using a vibratome (VT 1200S, Leica, Germany). Hippocampi were isolated from intact slices under a dissection microscope, and transferred onto Millicell culture inserts with 30 mm in diameter (EMD Millipore, MA, United States) with fresh Neurobasal medium containing 25% horse serum (HS) (Gibco, MA, United States), 22% HBSS, 1% D-glucose 550 mM, 1% L-glutamine 200 mM and 1% Antibiotic-Antimycotic below the membranes. Tissue slices were maintained at 37°C and 5% CO_2_ and the medium was renewed every 2 days. Experiments were performed at 14–15 DIV. One day before treatment, the medium was replaced by Neurobasal medium supplemented with 1% HS, 25% HBSS, 1.8% D-glucose 550 mM, 1% L-glutamine 200 mM and 1% Antibiotic-Antimycotic. For experiments with Aβ, slices were treated with 3 μM oAβ for 24 h.

### Western Blotting

Cell lysates were prepared from cultures using a cell scraper (Corning, NY, United States) and Pierce*™* RIPA buffer, with Halt*™* Protease and Phosphatase Inhibitor Cocktail (Thermo Fisher Scientific, MA, United States) and 0.5 M EDTA. Protein concentrations were determined by DC Protein Assay. Samples were diluted in 2 × sodium dodecyl sulfate (SDS) sample buffer and boiled at 95°C for 5 min before use. Equal amounts of protein lysates were loaded onto Bolt*™* 4–12% Bis-Tris mini protein gels, using Bolt*™* MES SDS Running buffer (Invitrogen, MA, United States) for electrophoresis, and transferred to iBlot*™* 2 PVDF membranes (Invitrogen, MA, United States), followed by immunoblotting. Membranes were blocked in 1 × tris–buffered saline (TBS) [20 mM Tris, 137 mM NaCl in double distilled water (ddH_2_O), pH 7.4], with 0.05% Tween−20 (TBS-T) (Acros organics, Belgium) and 5% fat-free milk for 1 h at room temperature (RT). Proteins were detected by incubation overnight with specific primary antibodies against PSD-95 (1:1,000, #ab18258, abcam, United Kingdom), VGlut1 (1:750, #135511, Synaptic Systems, Germany), Alpha tubulin (1:2,000, #ab7291, abcam, United Kingdom), MRC1 (1:1,000, #ab64693, abcam, United Kingdom), Arginase I (1:200, #sc-166920, Santa Cruz Biotechnology, CA, United States), Iba1 (1:500, #016-20001, Fujifilm Wako, Japan), CD11b (1:500, #ab128797, abcam, United Kingdom) and GAPDH (1:2,000, #MAB374, Sigma-Aldrich, MO, United States). Membranes were incubated with HRP-linked secondary antibodies anti-mouse IgG (1:5,000, #A6782, Sigma-Aldrich, MO, United States) and anti-rabbit IgG (1:5,000, #7074S, Cell Signaling Technology, MA, United States). Bands were detected with a ChemiDoc*™* MP Imaging System (Bio-Rad, CA, United States), and the band intensities were quantified using Bio-Rad Image Lab^®^ software. All protein intensities were divided by the corresponding tubulin or GAPDH measurement for normalization, unless otherwise stated.

### ELISA Assays

Pro-inflammatory cytokine Interleukin-1β (IL-1β) present in supernatants was measured using an appropriate ELISA kit (#ab100767, abcam, United Kingdom), following manufacturer’s instructions. Supernatant samples from cell cultures (density of 1 × 10^5^–1 × 10^6^ cells) were used undiluted. In brief, 100 μl of standards and samples were added into a 96-well plate pre-coated for rat IL-1β and incubated for 2.5 h at RT with gentle shaking. The plate was washed with washing solution, and 100 μl of Biotinylated IL-1β detection antibody were added to each well. After incubating for 1 h at RT with gentle shaking, the plate was washed again, and 100 μl of HRP-Streptavidin solution were added to each well. The plate was incubated for 45 min at RT with gentle shaking and washed before adding 100 μl of TMB One-Step substrate reagent to each well. After a 30 min incubation at RT with gentle shaking in darkness, 50 μl of Stop solution were added and the plate was read immediately in a fluorimeter at 450 nm. The standard curve was obtained plotting the standard concentration (pg/ml) on the *x*-axis and absorbance on the *y*-axis. IL-1β concentrations in each sample were calculated using the equation of the best-fit straight line through the standard points.

Levels of anti-inflammatory cytokine Transforming growth factor-β1 (TGF-β1) in supernatants were measured by an ELISA kit (#ab119558, abcam, United Kingdom), following manufacturer’s instructions. Amicon^®^ Ultracel^®^ 3K centrifuge filters (EMD Millipore, MA, United States) were used to concentrate the amount of proteins present in the cell culture supernatants (density of 1 × 10^5^–1 × 10^6^ cells), due to low levels of TGF-β1. For the ELISA assay, 20 μl of all samples were prediluted with 180 μl of Assay buffer plus 20 μl of 1N HCl for 1 h, and then neutralized with 20 μl 1N NaOH. 100 μl of standards and samples were added into a 96-well plate pre-coated for rat TGF-β1 and incubated for 2 h at RT with gentle shaking. The plate was washed with washing solution, and 100 μl of Biotin-Conjugated Antibody were added to each well. After incubating for 1 h at RT with gentle shaking, the plate was washed again, and 100 μl of Streptavidin-HRP solution were added to each well. The plate was incubated for 30 min at RT with gentle shaking and washed before adding 100 μl of TMB Substrate Solution to each well. After 30 min incubation at RT with gentle shaking in darkness, 100 μl of Stop solution were added and the plate was read immediately in a fluorimeter at 450 nm. The standard curve was obtained plotting the standard concentration (pg/ml) on the *x*-axis and absorbance on the *y*-axis. TGF-β1 concentration in each sample was calculated using the equation of the best-fit straight line through the standard points.

### Inmunofluorescence

Cell culture coverslips were fixed in 4% PFA for 10 min, and washed twice with 1 × TBS. Then, they were permeabilized and blocked in 1 × TBS with 1% BSA, 0.5% normal goat serum (NGS) (Palex Medical, Spain) and 0.1% saponin for 1 h. Coverslips were incubated overnight at 4°C with primary antibodies against MAP2 (1:1,000, #M9942, Sigma-Aldrich, MO, United States), Iba1 (1:500, #234004, Synaptic Systems, Germany), GFAP (1:1,000, #MAB3402, Sigma-Aldrich, MO, United States), Synaptophysin (1:1,000, #837101, BioLegend, CA, United States), Homer1 (1:500, #160003, Synaptic Systems, Germany), iNOS (1:200, #610329, BD Biosciences, NJ, United States), CD11b (1:500, #ab128797, abcam, United Kingdom), C3 (1:100, #ab11887, abcam, United Kingdom), PSD95 (1:500, #ab18258, abcam, United Kingdom), AMIGO2 (1:250, #sc-373699, Santa Cruz Biotechnology, CA, United States) and GFAP (1:4,000, #Z0334, Dako, CA, United States). Samples were washed with TBS-T and incubated with Alexa fluorophore-conjugated secondary antibodies (1:500, Invitrogen, MA, United States) for 1 h at RT. Cell nuclei were stained by incubating with 5 μg/ml DAPI (Sigma-Aldrich, MO, United States) for 15 min at RT, and samples were mounted using Fluoromont-G (SouthernBiotech, AL, United States).

Organotypic hippocampal slices were fixed in 4% PFA for 40 min, washed twice with 1 × phosphate-buffered saline (PBS) (125 mM NaCl, 19 mM Na_2_HPO_4_, 8 mM KH_2_PO_4_ in ddH_2_O, pH 7.4), and the membrane was trimmed around each slice with a scalpel for better handling. Samples were permeabilized and blocked for 3 h at RT in 1 × PBS with 5% NGS and 0.5% Triton-X. Then, slices were incubated overnight at 4°C in 1 × PBS with 1% NGS and 0.1% Triton-X with primary antibodies against Synaptophysin (1:1,000, #837101, BioLegend, CA, United States), PSD95 (1:500, #ab18258, abcam, United Kingdom) and CD11b (1:500, #MCA275R, Bio-Rad, CA, United States). Samples were washed with 1 × PBS and incubated with Alexa fluorophore-conjugated secondary antibodies (1:500, Invitrogen, MA, United States) for 2 h at RT. Slices were mounted using a bridge mounting technique (Yoon et al., 2010). Two 22 mm × 22 mm coverslips were glued to each end of a glass slide. Organotypic slices were mounted using Fluoromont-G, and a 40 mm × 22 mm coverslip was placed to bridge the gap without smashing the samples.

### Confocal Microscopy and Image Processing

Images were acquired using Leica TCS STED CW SP8X confocal microscope (Leica, Germany). In experiments with multiple fluorophores, channels were scanned sequentially to avoid crosstalk. The same settings were applied to all images within the same experiment. All analyses were carried out using open source ImageJ/Fiji software.

Synaptic markers were quantified in neuritic segments of primary neurons. In each condition, images (size 184 μm × 184 μm, resolution 5.55 pixels/μm) were acquired on random fields, and neuritic segments (20 μm in length) were selected from areas where a single process could be outlined. After the background was subtracted and a threshold was applied, integrated density of each neuritic segment was quantified. For each condition, individual segments were grouped and averaged per field, and all the per field averages were used to calculate the group mean and standard deviation for each condition and experiment. A macro was developed in ImageJ/Fiji software to automate the analysis.

For neuronal morphology analyses, MAP2^+^ positive cells were used. Single neurons were selected in areas where a single cell could be identified. Images were converted to binary and then skeletonized. The function Summarize skeleton in ImageJ/Fiji software was used to obtain the average branch length, the number of branches and the number of junctions in each neuron. The values of each individual cell were used to obtain the group mean and standard deviation for each condition and experiment.

To quantify the expression levels of proteins CD11b, iNOS, and C3 in the cell cultures, the specific markers MAP2, GFAP, and Iba1 were used to identify and define the area occupied by neurons, astrocytes and microglia, respectively. Then, the integrated density of each target protein was measured inside that delimited area. The integrated density of individual cells was averaged and divided by the total number of cells to obtain the integrated density per cell group mean and standard deviation for each condition and experiment.

To study astrocytic morphology *in vitro*, GFAP was used to stain astrocytes. They were selected in areas where a single astrocyte could be identified. After images of individual cells were transformed into binary, the tool Outline was used to automatically draw the cell shape. Morphology parameters like density (number of pixels of foreground color divided by the total number of pixels in the convex hull), span ratio (a measure of shape, as ratio of major and minor axes for the convex hull) and circularity were quantified using the Fractal Analysis FracLac plugin available in ImageJ/Fiji software as previously described (Young and Morrison, 2018). The values from individual cells were averaged to obtain the group mean and standard deviation for each condition and experiment.

For synaptic puncta colocalization studies, random fields in hippocampal organotypic slices were imaged with the 63× objective and 4× zoom in Leica TCS STED CW SP8X confocal microscope. Each image (size 34 μm × 34 μm, resolution 14.22 pixels/micron) was deconvoluted to better distinguish synaptic puncta using Huygens Professional software (Scientific Volume Imaging, Netherlands). The number of pre-synaptic, post-synaptic and colocalized puncta (spots where the two synaptic channels overlapped) was determined for each image. The values from each image were averaged to obtain the group mean and standard deviation for each condition and experiment. A macro was developed in ImageJ/Fiji software to automate the analysis.

### Statistical Analysis

GraphPad Prism 8 (GraphPad Software, CA, United States) was used to perform all statistical analyses. Data were presented as mean ± SEM (standard error of the mean) and scaled so that the average value for the corresponding control was 100%. Each independent experiment was coded with a different color. All data sets were tested for normality and homoscedasticity. Two-tailed paired Student’s *t*-test was used to compare two experimental groups. Comparisons between more than two groups were performed by paired one-way ANOVA, followed by *post-hoc* Tukey test. If one or more data points were missing, mixed-effect model was used to perform the analysis. Statistical significance was represented as *p* < 0.05 (*), *p* < 0.01 (^**^), and *p* < 0.001 (^***^).

## Results

### Neuron and Glia Triple Co-culture Model Optimization

In order to establish an *in vitro* protocol where we could study neuron-glia communication and recapitulate neurodegeneration features such as synaptic loss, we optimized an easy and straightforward murine triple co-culture model with neurons, astrocytes and microglia in all possible combinations (Figures 1A,B). We adapted the protocols for culturing individual murine neurons (Alberdi et al., 2018), astrocytes (Wyssenbach et al., 2016) and microglia (Zabala et al., 2018) to establish the triple co-culture model by identifying the appropriate platting order and ratios for the three cell types and by confirming their survival (Hernangómez et al., 2012; Correa et al., 2013). Plating astrocytes on top of neurons compromised neuronal survival (data not shown). Therefore, astrocytes where plated first and alone for 3 days to allow attachment and establishment of a monolayer. We identified the optimal ratio of astrocytes and neurons to be 2:5, since smaller proportions of neurons compromised neuronal survival and bigger proportions formed big clusters of cells not allowing individual quantification and characterization. Neurons were co-cultured with astrocytes for 7 days to allow maturation of neurons, following optimal time point established for neuronal individual primary cultures (Alberdi et al., 2018). Seven days after neurons were plated on top of astrocytes, microglia were plated on top of the astrocyte-neuron co-culture in a ratio of 2 astrocytes: 5 neurons: 1 microglia. Base medium for the triple co-culture consisted in serum-free neurobasal medium supplemented with B27 to ensure neuronal survival. Base medium did not compromise survival of astrocytes and microglia. FdU/U inhibitors of proliferation were included in the base medium for the maintenance of the optimal cellular ratio. Each cell type in the co-culture was characterized and compared with individual primary cultures as follows.

### Neurons Increase Post-synaptic Marker Expression and Develop a More Complex Morphology When Co-cultured With Glial Cells

Synapse formation and stabilization is promoted *in vitro* by astrocytes through secreted factors (Christopherson et al., 2005). Thus, we analyzed whether neurons would develop more synapses in the triple co-culture environment. To do so, we quantified pre- and post-synaptic markers in our co-cultures using western blotting (WB) and immunofluorescence (IF). We found that post-synaptic markers PSD-95 (30.0 ± 9.1%; *p* = 0.0408; *n* = 4) and Homer1 (34.5 ± 10.5%; *p* = 0.0093; *n* = 6) were increased in the triple co-culture compared with primary neurons alone (Figures 1C–F). Both microglia and astrocytes had to be present to observe this increase, because Homer1 was not altered in neuron-microglia or neuron-astrocyte co-cultures. Meanwhile, pre-synaptic markers VGlut1 (*p* = 0.8717; *n* = 4) and Synaptophysin (Syn) (*p* = 0.4053; *n* = 6) were not different in the triple co-culture compared to primary neurons (Figures 1G–J).

Studies carried out using dual co-cultures have suggested a change in neuronal morphology in the presence of microglia (Zhang and Fedoroff, 1996). Accordingly, we analyzed cellular morphology using the neuronal marker MAP2. In the triple co-culture, neurons displayed a higher number of branches (56.7 ± 23.3%; *p* = 0.0290; *n* = 4), as well as a higher number of junctions (62.6 ± 24.2%; *p* = 0.0190; *n* = 4). These branches were significantly longer (12.8 ± 11.5%; *p* = 0.0431; *n* = 4) compared with primary neurons alone (Figures 1K–N). It is worth noting that astrocytes alone showed a trend to increase the number of branches and junctions in neurons, although it was not statistically significant. In general, the conditions generated in the triple co-culture increased the number of post-synapses in neurons, as well as their cellular complexity.

### Microglia Display a Less Inflammatory Phenotype When Co-cultured With Neurons and Astrocytes

After observing that neurons exhibit different characteristics when glial cells were present, and given that microglial transcriptomic profiles are very dependent on the cellular environment (Schmid et al., 2009), we analyzed the possible phenotypic changes that microglia could undergo in our triple co-culture model.

**FIGURE 2:**
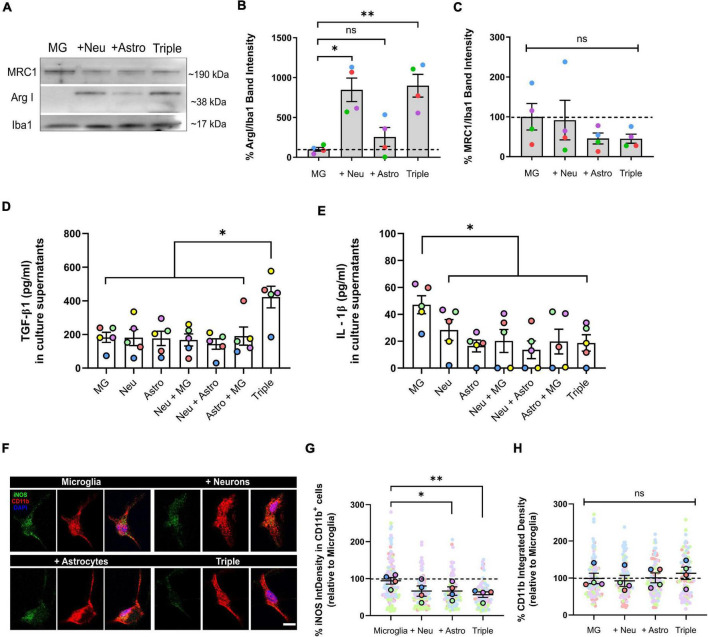
Microglia increased the expression of anti-inflammatory markers ArgI and TGF-β1, and decreased pro-inflammatory markers IL-1β and iNOS in the triple co-culture model. **(A)** Representative western blot image of microglial markers in total cell lysates. **(B)** Quantification of anti-inflammatory marker ArgI (*n* = 4). **(C)** Quantification of anti-inflammatory marker MRC1 (*n* = 4). Data was normalized using the marker Iba1 in order to selectively control for microglia. **(D)** ELISA quantification of anti-inflammatory cytokine TGF-β1 in culture supernatants (*n* = 5). **(E)** ELISA quantification of pro-inflammatory cytokine IL-1β in culture supernatants (*n* = 5). **(F)** Representative image microglial markers CD11b and iNOS in individual cells. Scale bar = 10 μm. **(G)** Quantification of the integrated density of iNOS inside CD11b^+^ cells (*n* = 4). **(H)** Quantification of the integrated density of CD11b (*n* = 4). Each bar represents the mean ± SEM. *p < 0.05; **p < 0.01; ns, not significant. For quantification, 5–6 random fields per condition were used. Neu, neurons; MG, microglia; Astro, astrocytes; ArgI, arginase I, IntDensity, integrated density.

We used WB, IF, and ELISA assays to quantify the expression of commonly used markers for pro- and anti-inflammatory microglial polarization. We found that the typically anti-inflammatory marker Arginase I (ArgI) displayed a ∼ 8-fold increase in the triple co-culture (798.2 ± 142.7%; *p* = 0.0072; *n* = 4), as well as in the microglia-neuron co-culture (746.2 ± 147.3%; *p* = 0.0157; *n* = 4) compared with primary microglia alone (Figures 2A,B). No changes were found in the case of anti-inflammatory marker Mannose receptor C-type 1 (MRC1) (*p* = 0.0899; *n* = 4) (Figures 2A,C). We also found a ∼ 2-fold increase of the anti-inflammatory cytokine TGF-β1 in triple co-culture supernatants compared with supernatants from primary microglia (422.2 ± 64.4 pg/ml vs. 182.7 ± 32.2 pg/ml; *p* = 0.0067; *n* = 5), primary neurons (422.2 ± 64.4 pg/ml vs. 181.5 ± 47.6 pg/ml; *p* = 0.0026; *n* = 5) and primary astrocytes (422.2 ± 64.4 pg/ml vs. 176.7 ± 42.1 pg/ml; *p* = 0.0093; *n* = 5) (Figure 2D). On the other hand, microglia decreased the secretion of pro-inflammatory cytokine IL-1β in the triple co-culture compared to microglia alone (18.6 ± 6.1 pg/ml vs. 47.1 ± 6.7 pg/ml; *p* = 0.0059; *n* = 5) (Figure 2E). At the same time, there was a decrease of the pro-inflammatory marker iNOS in the triple co-culture (39.8 ± 7.6%; *p* = 0.0085; *n* = 4) and in the astrocyte-microglia co-culture (29.5 ± 11.3%; *p* = 0.0378; *n* = 4) compared with primary microglia alone (Figures 2F,G). No changes were observed in microglial activation marker CD11b (*p* = 0.3087; *n* = 4) (Figures 2F,H). In conclusion, microglia had a less inflammatory phenotype in the triple co-culture model compared with primary culture conditions, revealed by an increase of anti-inflammatory markers ArgI and a decrease of pro-inflammatory markers IL-1β and iNOS. Also, the increase of TGF-β1 in the triple-co-culture model compared to the individual cell cultures, supports the contribution of different cell types to the overall anti-inflammatory profile of the co-cultured.

### Astrocytes Turn to a Less Reactive Phenotype in the Triple Co-culture Model

Once we observed that microglia were less inflammatory in the triple co-culture, we addressed astrocytic activation in the model. Besides, reactive microglia are necessary to induce A1 reactive astrocytes *in vitro via* secreted signaling molecules (Liddelow et al., 2017).

**FIGURE 3:**
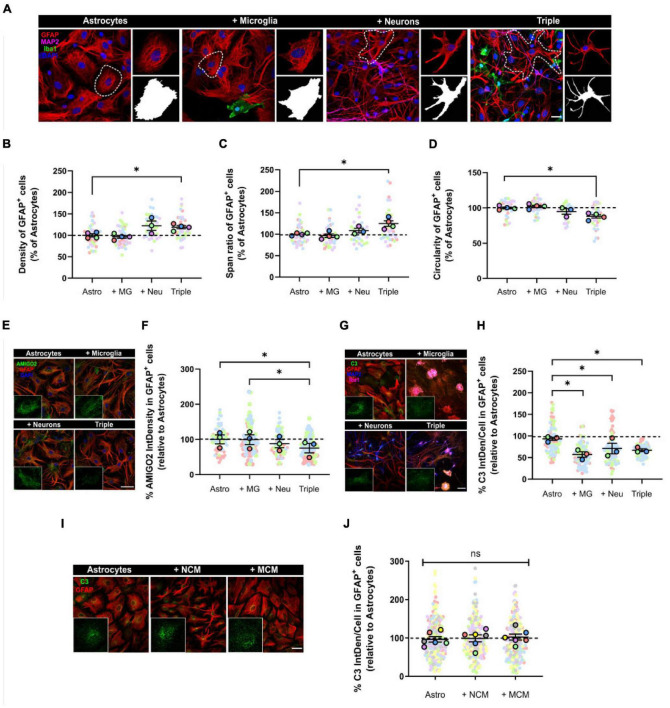
Astrocytes displayed a less reactive morphology and reduced expression of A1 activation markers AMIGO2 and C3. **(A)** Representative images of the analysis of astrocytic morphology using GFAP and Fractal Analysis in Fiji Software. Black and white images represent the outline of individual cells selected in each condition. Scale bar = 20 μm. **(B)** Quantification of the density of GFAP^+^ astrocytes (*n* = 4). **(C)** Quantification of the span ratio of GFAP^+^ astrocytes (*n* = 4). **(D)** Quantification of the circularity of GFAP^+^ astrocytes (*n* = 4). **(E)** Representative image of A1 astrocytic marker AMIGO2. Scale bar = 40 μm. **(F)** Quantification of the expression of AMIGO2 inside GFAP^+^ cells (*n* = 3). **(G)** Representative image of A1 astrocytic marker C3. Scale bar = 40 μm. **(H)** Quantification of the integrated density of C3 inside GFAP^+^ cells (*n* = 3). **(I)** Representative image of C3 in GFAP^+^ astrocytes after conditioned media treatment. Scale bar = 40 μm. **(J)** Quantification of the integrated density of C3 (*n* = 6). Each bar represents the mean ± SEM. *p < 0.05; ns, not significant. For quantification, 5–6 random fields per condition were used. Neu, neurons; MG, microglia; Astro, astrocytes; NCM, neuron conditioned media; MCM, microglia conditioned media; IntDen, Integrated density.

Firstly, we analyzed astrocytic morphology as a measure of their activation state. We used the astrocytic marker GFAP to obtain morphological parameters. We observed that, in the triple co-culture, astrocytes significantly increased the parameters of density (18.9 ± 3.7%; *p* = 0.0292; *n* = 4) and span ratio (24.9 ± 6.4%; *p* = 0.0401; *n* = 4), while they reduced their circularity (11.9 ± 2.3%; *p* = 0.0123; *n* = 4) compared with primary astrocytes (Figures 3A–D). These parameters in the triple co-culture reflect an increase in cellular ramifications, which resembles better the physiological resting state. The presence of neurons was enough to see a morphological change in astrocytes, although it was not statistically significant.

We also quantified the expression of the two A1 astrocytic markers AMIGO2 and C3, using IF. In the triple co-culture, GFAP^+^ astrocytes significantly decreased the expression of AMIGO2 compared with microglia-astrocyte co-culture (23.9 ± 13.4%; *p* = 0.0323; *n* = 3) and primary astrocytes (24.6 ± 13.4%; *p* = 0.0387; *n* = 3) (Figures 3E,F). In addition, C3 expression in astrocytes decreased in all of the co-culture conditions; in the microglia-astrocyte co-culture (38.7 ± 6.4%; *p* = 0.0296; *n* = 3), in the neuron-astrocyte co-culture (23.8 ± 12.3%; *p* = 0.0343; *n* = 3) and in the triple co-culture (28.0 ± 3.3%; *p* = 0.0376; *n* = 3) (Figures 3G,H). In order to analyze if astrocytic C3 was modulated *via* cell-to-cell contact or *via* secreted factors, we used conditioned media. We incubated primary astrocytes with conditioned media from neurons (NCM) or from microglia (MCM) for 3 days, and then quantified C3. There were no changes in C3 levels with either NCM (*p* = 0.6454; *n* = 6) or MCM (*p* = 0.6897; *n* = 6), so physical contact between cells was required for astrocytes to reduce C3 expression (Figures 3I,J). In conclusion, astrocytes seemed less reactive in the triple co-culture environment, with a more ramified morphology and reduced expression of A1 markers AMIGO2 and C3.

### Oligomeric Aβ Induces Synaptic Loss and Increases Microglial CD11b in the Triple Co-culture

Once we characterized the triple co-culture, we used it to establish a model where we could study AD *in vitro*, without the limitations of single primary cultures. We used oAβ as the stimulus to induce neurodegeneration, since oligomers seem to be the most neurotoxic amyloid species (as reviewed in Glabe, 2006).

**FIGURE 4:**
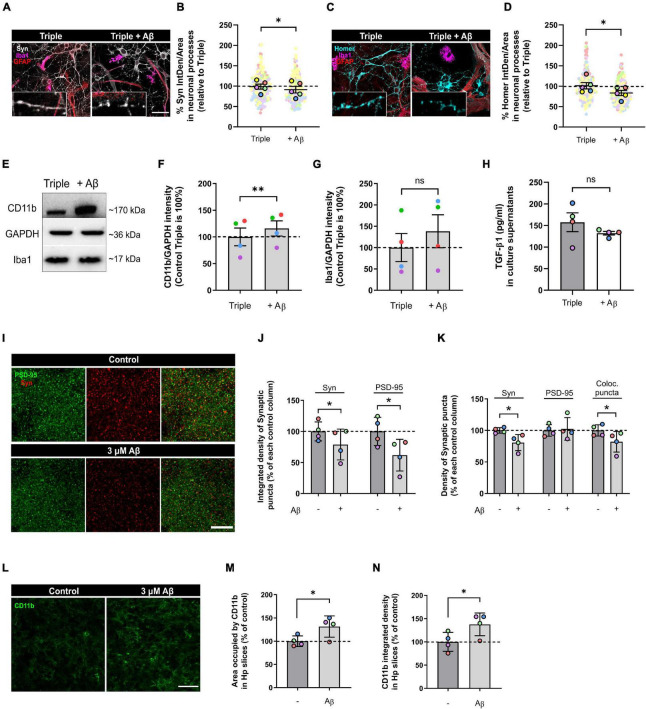
Oligomeric Aβ reduced pre- and post-synaptic puncta and increased microglial expression of CD11b in the triple co-culture and in hippocampal organotypic slices. **(A)** Representative image of Synaptophysin in neuronal processes. Scale bar = 40 μm. **(B)** Quantification of Synaptophysin in the triple co-culture after oAβ treatment (*n* = 5). **(C)** Representative image of Homer1 in neuronal processes. Scale bar = 40 μm. **(D)** Quantification of Homer1 in the triple co-culture with oAβ (*n* = 5). **(E)** Western blot image of microglial markers in total lysates. **(F)** Quantification of CD11b in the triple co-culture (*n* = 4). **(G)** Quantification of Iba1 expression (*n* = 4). Raw data was normalized with GAPDH. **(H)** ELISA quantification of TGF-β1 in culture supernatants (*n* = 4). **(I)** Representative images of synaptic markers puncta in hippocampal organotypic slices. Scale bar = 10 μm. **(J)** Integrated density of Synaptophysin and PSD-95 in organotypic cultures after oAβ treatment (*n* = 4). **(K)** Quantification of the puncta density of Synaptophysin, PSD-95 and the colocalization of both markers (*n* = 4). **(L)** Representative image of CD11b staining in hippocampal organotypic slices. Scale bar = 40 μm. **(M)** Area occupied by CD11b staining after oAβ treatment (*n* = 4). **(N)** Integrated density of CD11b (*n* = 4). Each bar represents the mean ± SEM. *p < 0.05; **p < 0.01; ns, not significant. For quantification, 5–6 random fields and 4 random fields per condition were quantified in triple co-cultures and organotypic cultures, respectively. Syn, synaptophysin.

We treated our triple co-cultures with 3 μM oAβ for 24 h, and then analyzed neurodegeneration features such as synaptic loss and neuroinflammation. We quantified pre- and post-synaptic markers in the neuronal processes using IF and found that both Syn and Homer1 were reduced with the oAβ treatment (7.6 ± 8.1%; *p* = 0.0364; *n* = 5) and (17.5 ± 6.4%; *p* = 0.0263; *n* = 5), respectively, compared with controls (Figures 4A–D). Along with synapse loss, neuroinflammation characterized by microglial CD11b increase is another key feature of Aβ-induced neurodegeneration *in vitro* and *in vivo* (Akiyama and McGeer, 1990; Jana et al., 2008; Zhang et al., 2011; Salih et al., 2019). For that reason, we analyzed CD11b in total lysate samples with WB, as a measure of microglia-related inflammation. We observed that, while Iba1 expression did not change (*p* = 0.1968; *n* = 4), CD11b increased in microglia with the oAβ treatment (16.0 ± 14.3%; *p* = 0.0067; *n* = 4) compared with controls (Figures 4E–G). Besides, there was a trend to decrease of anti-inflammatory cytokine TGF-β1 levels in the triple co-culture, although not statistically significant (*p* = 0.3709; *n* = 4) (Figure 4H).

To confirm our findings in the triple co-culture using a model with higher complexity, we used hippocampal organotypic cultures. We treated these organotypic cultures with the same conditions (3 μM oAβ for 24 h) and analyzed synaptic loss and neuroinflammation. Aβ treatment reduced the integrated density of Syn^+^ pre-synaptic puncta (21.1 ± 12.4%; *p* = 0.0470; *n* = 4) and PSD-95^+^ post-synaptic puncta (38.2 ± 12.8%; *p* = 0.0203; *n* = 4) compared with controls (Figures 4I,J). The puncta density was also decreased in the case of Syn (19.3 ± 6.4%; *p* = 0.0135; *n* = 4), which caused a reduction in the overall colocalization puncta (18.0 ± 8.1%; *p* = 0.0494; *n* = 4) (Figure 4K). Regarding neuroinflammation, we quantified CD11b^+^ microglia in the organotypic slices. We found that oAβ increased both the area occupied by CD11b (31.5 ± 11.4%; *p* = 0.0309; *n* = 4) and its integrated density (37.7 ± 12.3%; *p* = 0.0323; *n* = 4) compared to controls (Figures 4L–N). In conclusion, we were able to recapitulate Aβ-induced synaptic loss and neuroinflammation in our triple co-culture, and we confirmed these findings using hippocampal organotypic slices.

## Discussion

**FIGURE 5:**
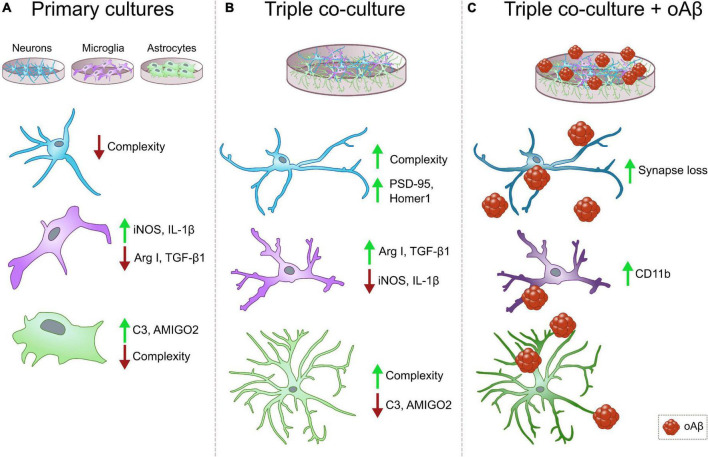
Summary diagrams of individual vs. triple co-cultures and in the context of AD. **(A)** Classical individual primary cultures often exhibit significant alterations in comparison with cells in physiological conditions. Neurons and microglia display low morphological complexity, and both microglia and astrocytes increase the expression of pro-inflammatory markers iNOS, IL1β and C3, and AMIGO2, respectively. **(B)** In our triple co-culture, neurons develop more complex morphologies and increased post-synaptic markers PSD-95 and Homer1. Microglia lower their expression of pro-inflammatory markers such as iNOS and IL-1β, and increase anti-inflammatory markers Arg I and TGF-β1. Lastly, astrocytes reduce the expression of pro-inflammatory markers C3 and AMIGO2, while they develop ramifications that resemble physiological conditions. **(C)** In the triple co-culture, Aβ1-42 oligomers are able to induce synaptic loss and increase microglial marker CD11b, which makes this triple co-culture a suitable model to study amyloid pathology associated changes *in vitro*. Neurons, blue cells, microglia, purple cells, astrocytes, green cells, oAβ, oligomeric Aβ.

We developed a murine triple co-culture model including neurons, microglia and astrocytes, that holds physiological characteristics that are lost in classical primary cultures. Microglia are less inflammatory, astrocytes are less reactive, and neurons display a more mature morphology than in individual primary cultures. With this model, we were able to recapitulate Aβ-induced AD pathological features like synaptic loss and CD11b increment, which will allow us to study neurodegeneration and neuroinflammation processes relevant to AD progression (Figure 5).

In our triple co-culture, neurons developed a higher number of post-synapses, while pre-synapses remained unchanged. This finding would align with previous work indicating that synaptogenesis promoted by astrocyte-secreted factors such as thrombospondins formed structurally normal synapses (Christopherson et al., 2005). Our model would allow further functional studies of neuronal activity, as well as the identification of glial factors with potential synaptogenic activity. We also observed that the presence of both microglia and astrocytes in the culture promoted a significant morphological change in neurons, which developed a higher number of branches that were also longer. These findings, along with previous studies demonstrating that the presence of astrocytes increases neuronal viability *in vitro* (Aebersold et al., 2018), support our co-culture as a relevant system to study neuronal function in AD and other neurodegenerative diseases.

Microglial genomic and proteomic profile is very dependent on the environment. Previous studies have shown a differential gene expression in primary microglia as opposed to the adult CNS, revealed by a dedifferentiation of microglia in culture (Schmid et al., 2009). The use of serum as a way to stimulate cell proliferation and survival *in vitro* also causes relevant phenotypic changes in microglia. Bohlen et al. (2017) demonstrated that serum induced significant alterations in microglial morphology and intrinsic phagocytic capacity, and that these alterations could be avoided using serum-free astrocyte conditioned media containing CSF-1/IL-34, TGF-β2, and cholesterol. In the case of our triple co-culture model, we used serum-free neurobasal medium, which allowed us to avoid serum-related changes while maintaining neuronal viability. In these serum-free co-culture conditions, microglia decreased the expression of pro-inflammatory markers iNOS and IL-1β while increased the expression of anti-inflammatory marker Arginase I. Anti-inflammatory cytokine TGF-β1 was also reduced. We consider that both the serum-free conditions and the presence of neurons and astrocytes, contributed to maintain microglia in a less reactive state compared with primary cultures.

In our model, we observed that, microglia being less reactive, astrocytes had a ramified morphology and decreased the expression of A1 activation markers AMIGO2 and C3. We did not observe any expression changes in astrocytic C3 with conditioned media. Liddelow et al. (2017) reported that condition media from LPS activated microglia, but not from non-activated microglia, strongly induced reactive A1 astrocytes (Liddelow et al., 2017). Our results go in line with these observations since under physiological conditions, without any external inflammatory stimulus such as LPS or oligomeric Aβ, only the soluble factors present in the microglial condition media are not enough to modified astrocytic C3 expression. However, our results seem to contradict a previous report that, using a tri-culture system with hPSC-derived microglia, astrocytes, and neurons, found an increase in astrocytic C3 in the presence of microglia and microglia conditioned media (Guttikonda et al., 2021). This discrepancy could be because the authors quantified secreted C3, while we quantified C3 within astrocytes. Also, both platting rates and the incubation time that cells and/or condition media were in contact with each other differ between our murine triple co-culture and the one derived from hPSCs, parameters that could also lead to different cellular responses. Thus, a more exhaustive analysis will be necessary to understand the communication processes between cell types.

Our model is complementary to existing triple models of neuroinflammation, and displays certain advantages. The morphological changes that we observed in astrocytes were also reported in a previous publication that used rat triple co-cultures to model LPS and injury-induced neuroinflammation. In this publication, the triple co-culture was obtained from a pool of dissociated cortex tissue (Goshi et al., 2020). In our case, we first cultured each cell type separately, which allows a better control over the number of seeded cells. A recently published triple co-culture model of LPS-induced neuroinflammation used immortalized cell lines in transwells, which allowed control over cell numbers, but did not allow physical contact between the cells (Zheng et al., 2021). In our model, physical interaction between cells can be studied and compared with conditioned media. More advanced triple co-cultures use human PSCs. Guttikonda et al. (2021) developed a triple co-culture using PSC-derived neurons, astrocytes and microglia carrying the APP Swedish mutation to study complement component C3 regulation by glial cells *via* reciprocal signaling (Guttikonda et al., 2021). Park et al. (2018) also used human PSCs with AD mutations to establish a 3D triple co-culture model in which they studied microglia migration and neurotoxicity. These models might have the advantage of closer recapitulating AD pathology from patients compared to ours since their cellular source is human. However, human PSCs maintenance and 3D culture setups are methodologically more difficult and require more resources than murine cultures. Besides, the differentiation of each cell type is very time consuming, most of the time taking up to months. In comparison, our murine triple co-culture is easy to implement because it does not require sophisticated setups and takes reasonably short times, while it still allows to study AD pathology and cellular interactions.

We used oligomeric Aβ to model AD conditions. Exogenous addition of Aβ has been widely used in primary cultures to study amyloid pathology. Both Aβ oligomers and fibrils reduce neuronal viability (Ryan et al., 2010; Janefjord et al., 2014), and Aβ oligomers induce alterations in synaptic spine numbers and morphology in primary neurons (Calabrese et al., 2007; Lacor et al., 2007). In primary astrocytes, Aβ oligomers can reduce cell viability and increase activation markers (Hou et al., 2011; Alberdi et al., 2013). Aβ oligomers also increase pro-inflammatory cytokine release (Li et al., 2013) and activate the inflammasome in microglia cultures (Lučiūnaitë et al., 2020). Microglial CD11b increase has been reported in AD patients (Akiyama and McGeer, 1990) and it has been associated as potential risk gene (Salih et al., 2019). Severity of microglial activation is reflected by the increased on CD11b expression in LPS induced inflammation (Roy et al., 2008). In our triple co-culture, addition of exogenous Aβ oligomers induced pre- and post-synaptic loss, as well as an increase of neuroinflammation reflected by an increase in CD11b marker. Guttikonda et al. (2021) reported that astrocytes secrete high levels of C3 upon LPS stimulation in the triple AD co-culture derived from human PSC. LPS might induced stronger inflammatory response than 3 μM oligomeric Aβ, but interestingly our results are complementary to those ones, since we report high levels of CD11b, one of the subunits of the CR3, receptor for cleavage products of C3. Both microglia and astrocytes can participate in synaptic elimination *via* the complement system (Hong et al., 2016) and MEGF10/MERTK pathways (Chung et al., 2013), respectively. Therefore, our model could be useful to further study the role of these pathways in Aβ-induced synaptic loss. We used a more complex model such as hippocampal organotypic cultures to confirm our findings and observed the same negative effects of oligomeric Aβ on synaptic loss and neuroinflammation. Compared with organotypic cultures, our triple co-culture model has a defined concentration of each cell type that can be manipulated, becoming a complementary model for mechanistic studies.

Altogether, our findings suggest that the neuron, microglia, and astrocyte triple co-culture model we developed is reliable, affordable and a useful tool for the study of mechanisms implicated in AD neurodegeneration. In agreement with previous reports with *in vitro* models, ours also closely mimics the *in vivo* environment, where the cross talk between neurons and glial cells contributes to neuroinflammation processes (Goshi et al., 2020), and where alterations in glial cells can lead to neurodegeneration (Lange et al., 2018). Our model can also be potentially used in high throughput screening setups, so we expect that it will help finding new therapeutic targets for AD and other neurodegenerative diseases.

## Data Availability Statement

The raw data supporting the conclusions of this article will be made available by the authors upon request, without undue reservation.

## Ethics Statement

This study was reviewed and approved by the Animal Ethics and Welfare Committee of the University of the Basque Country (UPV/EHU), following the Spanish Real Order 53/2013 and the European Communities Council Directive of September 22nd 2010 (2010/63/EU).

## Author Contributions

EC-Z and CL conceived the experiments and wrote the manuscript. CL and JZ-I performed the experiments. JV provided tools for image analysis. EA and CM provided valuable insight and advice throughout the project. All authors read and approved the final manuscript.

## Conflict of Interest

The authors declare that the research was conducted in the absence of any commercial or financial relationships that could be construed as a potential conflict of interest.

## Publisher’s Note

All claims expressed in this article are solely those of the authors and do not necessarily represent those of their affiliated organizations, or those of the publisher, the editors and the reviewers. Any product that may be evaluated in this article, or claim that may be made by its manufacturer, is not guaranteed or endorsed by the publisher.
